# 578. Performance of acid-fast stain, real-time PCR, and Xpert MTB/RIF in a bronchial washing for the Rapid Diagnosis of Mycobacterial infection in real-world

**DOI:** 10.1093/ofid/ofad500.647

**Published:** 2023-11-27

**Authors:** Joo-Hee Hwang, Jeong-Hwan Hwang, Sung-Sik Choi, Hyo-Jin Han, Seung Yeob Lee, Joonhong Park, Dae Sun Jo, Yong Gon Cho, Dal Sik Kim, Jaehyeon Lee

**Affiliations:** Jeonbuk National University Medical School and Hospital, Jeonju, Cholla-bukto, Republic of Korea; Jeonbuk National University Medical School and Hospital, Jeonju, Cholla-bukto, Republic of Korea; Jeonbuk National University Medical School and Hospital, Jeonju, Cholla-bukto, Republic of Korea; Jeonbuk National University Medical School and Hospital, Jeonju, Cholla-bukto, Republic of Korea; Jeonbuk National University Medical School and Hospital, Jeonju, Cholla-bukto, Republic of Korea; Jeonbuk National University Medical School and Hospital, Jeonju, Cholla-bukto, Republic of Korea; Jeonbuk National University Medical School and Hospital, Jeonju, Cholla-bukto, Republic of Korea; Jeonbuk National University Medical School and Hospital, Jeonju, Cholla-bukto, Republic of Korea; Jeonbuk National University Medical School and Hospital, Jeonju, Cholla-bukto, Republic of Korea; Jeonbuk National University Medical School and Hospital, Jeonju, Cholla-bukto, Republic of Korea

## Abstract

**Background:**

Mycobacterium culture remains still the gold standard for diagnosing mycobacterium infections, but there are some limitations. Acid-fast stain and simultaneous molecular methods are recommended for early diagnosis and treatment. Bronchial washing has been reported to be an effective diagnostic sample, even in cases sputum smear tests are negative. Additionally, it can be used in multiple assays. Therefore, we analyzed the clinical performance of acid-fast stain, real-time PCR, and Xpert MTB/RIF assay compared to mycobacterial cultures in the same bronchial washing.

**Figure:**
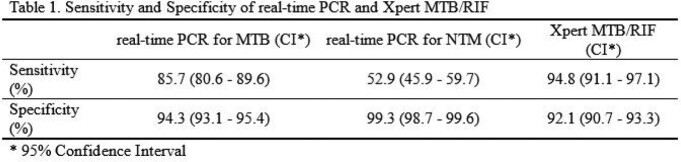

**Methods:**

We screened laboratory data from January 2018 to December 2020 at Jeonbuk National University Hospital. We enrolled the samples that all the tests for analysis, acid-fast stain, rPCR, Xpert MTB/RIF, and mycobacterial culture, were ordered simultaneously. The study was approved by the Institutional Review Board of Jeonbuk National University Hospital (CUH 2022-05-036-001). In short, the bronchial washing was sent to a clinical microbiology laboratory. The cultures were done with 2% Ogawa medium and BacT/Alert MB culture bottle after NALC-NaOH. The rPCR and the Xpert MTB/RIF was done according to the manufacturer's guide. The statistical analysis was done with Excel 2016 and Analyse-it.

**Results:**

A total of 1966 samples were enrolled, and the culture-positive sample is defined when either the solid or liquid cultures showed positive. In the mycobacterium culture, 12.8% were MTB positive, and 10.7% were non-tuberculosis Mycobacterium (NTM) positive. The sensitivity and specificity of acid-fast stain were 45.6% and 98.9%, respectively, compared to mycobacterium culture positive, including MTB or NTM. The sensitivity and specificity of rPCR in MTB and NTM are 85.7%/94.3% and 52.9%/99.3%, respectively, and Xpert MTB/RIF in MTB cases was 94.8% and 92.1% (Table 1).

**Conclusion:**

The acid-fast stain is still a valuable diagnostic tool despite its low sensitivity because it is easy to prepare and it doesn't require specialized equipment or facilities. The rPCR for MTB showed reliable results. However, in rPCR for NTM, the sensitivity was much lower than MTB. Xpert MTB/RIF showed superior sensitivity than rPCR here. The assays should be used strategically in suspected mycobacterium infections.

**Disclosures:**

**All Authors**: No reported disclosures

